# Influence of thyroidectomy on postoperative serum calcium level regarding serum vitamin D status. A prospective study

**Published:** 2015

**Authors:** Gholamali Godazandeh, Zahra Kashi, Farnaz Godazandeh, Pouya Tayebi, Ali Bijani

**Affiliations:** 1 Department of Thoracic Surgery, Imam Khomeini Hospital, Mazandaran University of Medical Sciences, Sari, Iran.; 2 Diabetes Research Center, Mazandaran University of Medical Sciences, Sari, Iran; 3 Department of Radiology, Imam Khomeini Hospital , Mazandaran University of Medical Sciences, Sari, Iran.; 4 Social Determinant of Health Research Center, Babol University of Medical Sciences, Babol, Iran.

**Keywords:** Hypocalcemia, Transient hypocalcemia, Vitamin D deficiency, Thyroidectomy, Hypoparathyroidism

## Abstract

**Background:**

Hypocalcemia is a well-recognized complication after total thyroidectomy. Hypovitaminosis D may have additional effect in the development of hypocalcemia. This study aimed to determine the effect of total thyroidectomy on postoperative serum calcium in patients with and without hypovitaminosis D.

**Methods:**

This prospective study was performed on patients who underwent total thyroidectomy from 2011 to 2014 in Imam Khomeini General Hospital of Mazandaran University of Medical Sciences. Serum calcium and vitamin D values were recorded before and after surgery. The patients were classified according to serum vitamin D concentrations as less 10 ng/ml (vitamin D deficiency) or higher (control group). The mean values of postoperative calcium level for each class of serum vitamin D were determined and compared. Hypocalcemia was defined as a postoperative calcium level <8 mg/dl.

**Results:**

125 patients due to thyroid disease underwent total thyroidectomy. The incidence of symptomatic and asymptomatic hypocalcemia after surgery was 12% (n=15) and 3.2% (n=4) respectively. 82 (65.6%) patients had vitamin D deficiency and 43 (34.4%) patients had sufficient vitamin D level. There was not any significant difference in calcium level (8.67±0.58 mg/dl vs. 8.70±0.59 mg/dl) between two vitamin D studied groups after thyroid surgery (p>0.05).

**Conclusion:**

The findings of this study indicated that vitamin D deficiency had no significant effect on post-thyroidectomy serum calcium level.

Hypocalcemia is the most common complication after thyroid surgery (1, 2). Numerous studies have reported the prevalence between 1.6-50% for transient hypocalcemia after total thyroidectomy (3-8). The clinical symptoms appearing in this situation are variable and include; paresthesia around the lips and tips of fingers, carpopedal spasm, convulsions, cardiac arrhythmias and laryngospasm (3, 9). Most cases of hypocalcemia are transient but permanent hypocalcemia can occur after 1.5% to 4% of surgeries that can be due to hypoparathyroidism (5, 10, 11). We know that vitamin D plays an important role in calcium homeostasis in human body. 

1, 25-dihydroxyvitamin D3 facilitates intestinal calcium absorption, whilst both 1, 25-dihydroxyvitamin D3 and PTH stimulate calcium release from bone. PTH also stimulates the conversion of 25-hydroxyvitamin D3 to 1,25-dihydroxyvitamin D3 enabling distal renal tubular calcium reabsorption (12). In a number of recent studies, preoperative serum levels of vitamin D have been introduced as factors affecting the incidence of postoperative hypocalcemia (4, 13). Unfortunately, vitamin D deficiency is prevalent in northern Iran. A study done by Kashi et al. (14) in northern Iran, vitamin D deficiency in winter was 88.9% and 71.4% at the end of summer. Due to low levels of vitamin D in northern Iran which can be as an important cause of hypocalcemia in patients suffering from hypocalcemia after thyroid surgery and also because of longer stay in the hospital, frequent tests and more treatments resulting to the average cost of an inpatient hospital stay increase (1, 2, 15, 16). We designed this study to evaluate the role of serum vitamin D levels before thyroidectomy in the incidence of postoperative hypocalcemia.

## Methods

This prospective study was followed-up at the Imam Khomeini Hospital of Mazandaran University of Medical Sciences 2011 to 2014. All patients who underwent total thyroidectomy have been included. The demographic and pathologic data of all the patients with their serum calcium and vitamin D levels before and after surgery were recorded. The patients with albumin less than 3.5 gr/dl, kidney disease (creatinine above 2 mg/dl), gastrointestinal disease (cirrhosis), and those with concomitant thyroidectomy and neck node dissection were excluded. Before surgery, all patients were adequately informed about the type of their surgery and its possible complications. The study was approved by the Ethics Committee of Mazandaran University of Medical Sciences. All surgeries were performed by same surgical team with extra capsular total thyroidectomy method and the Liga Sure (Covidien Force Triad,) was used for homeostasis. If any of the parathyroid glands were removed inadvertently, it was planted in sternocleidomastoid muscle immediately.

On the first and second day after surgery, the serum calcium level was measured separately. If the clinical signs and symptoms of hypocalcemia occurred or that serum calcium was less than 8 mg/dl, the treatment for calcium replacement was started. In the case of biochemical hypocalcemia treatment with oral calcium (500 mg-1gr ) three times a day along with oral calcitriol twice a day, and in clinically presented symptoms of hypocalcemia, treatment with intravenous calcium plus oral Calcitriol was started. The patients without biochemical or clinical hypocalcemia were discharged on the second day after surgery and was followed-up as an outpatient setting. 

The patients with hypocalcemia were discharged after the correction of serum calcium with oral calcium and Calcitriol. Serum 25-hydroxy vitamin D was also measured by Elecsys (Hitachi, Japan) machine and Roshe (Germany) Keith. The vitamin D values ​​less than 10 ng/ml were considered as deficient, and more than 10 ng/ml was considered as non-deficient control group (17). Also, serum calcium was measured by Hitachi 911 (Japan) machine with pars Keith. A serum calcium value less than 8 mg/dl is considered as hypocalcemia.

For the statistical analysis, the patients were divided into two categories based on their vitamin D serum levels before surgery: (1) vitamin D deficiency group with the amount of vitamin D less than 10 ng/ml and (2) control group with the amount of vitamin D higher than 10 ng/ml. T- test and repeated measures ANOVA were used for data analysis using SPSS statistical software. A p-value <0.05 was considered statistically significant.

## Results

**Fig 1:**
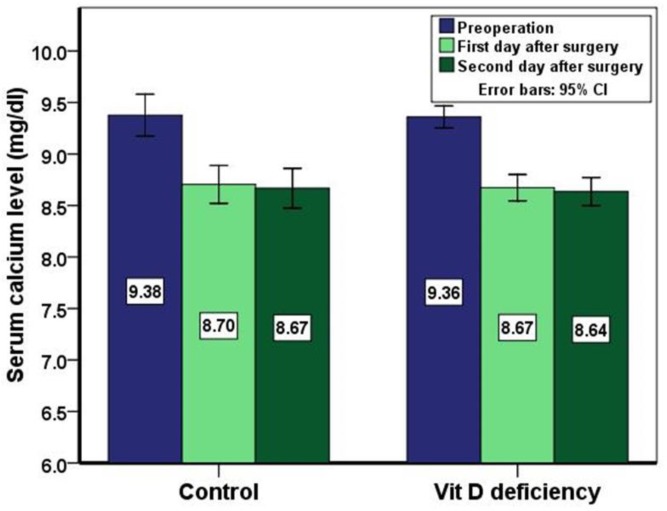
Serum calcium level (mean±SE) before surgery and on the first and second day after surgery in vitamin D studied groups.

In this study, 125 patients (104 females and 21 males) due to thyroid disease underwent total thyroidectomy. The mean age of patients was 42.02±13.66 years. 82 (65.6%) patients had vitamin D deficiency (vitamin D < 10 ng/ml) and 43 (34.4%) patients with higher value of vitamin D level (control group). The incidence of symptomatic hypocalcemia was 12% (n=15) and asymptomatic hypocalcemia (only the laboratory hypocalcemia; Ca<8 mg/dl) was 3.2% (n=4). Table 1 presents the values of serum calcium before and after thyroidectomy according to serum vitamin D levels. As shown in figure 1, the mean of serum calcium after thyroidectomy decreased in both vitamin D studied groups but these differences were not statistically significant in any of these groups (p>0.05). 

In other words, our findings indicated that the serum vitamin D level before surgery has no role in the incidence of postoperative calcium changes.

**Table 1:** Serum calcium level (mg/dl) before surgery and on the first and second day after surgery in vitamin D studied groups.

**Vitamin D** **Studied Groups**	**Serum calcium level (mean±SE)**			
	**Preoperation**	**1st day after operation**	**2nd day after operation**	
Deficient (n=82)	9.36±0.05	8.67±0.06	8.64±0.06
Control* (n=43)	9.37±0.10	8.70±0.09	8.67±0.09

* : Vitamin D >10ng/ml

## Discussion

Hypocalcemia is the most common complication after thyroidectomy (3-8). The role of vitamin D in the homeostasis of body calcium is important but despite vitamin D deficiency being common in patients undergoing thyroidectomy, our results do not suggest that this increases the rate of hypocalcemia. The findings of this study indicated that hypovitaminosis D has no effect on serum calcium level after thyroid surgery. Eighty two (65.6%) patients who underwent thyroid surgery in the present study had vitamin D deficiency preoperatively. In comparison with the control group, there was not any significant difference in the mean of calcium level (8.67±0.58 mg/dl vs. 8.70±0.59 mg/dl) between these studied groups after thyroid surgery (p>0.05). This finding indicates that vitamin D deficiency does not have a significant effect on post-thyroidectomy induced hypocalcemia. In contrast to our findings James-Krikby et al. in a prospective study on 166 patients who underwent total thyroidectomy have shown significant difference in the incidence of hypocalcemia after surgery for the patients with vitamin D > 50nmol/l (20ng/ml) and the patients with vitamin D <25nmol/l (<10ng/ml). This issue was associated with delayed patient's discharge because of hypocalcemia treatment (4). Also, Erbil et al. showed that vitamin D before surgery was the only significant independent variable that can affect the incidence of hypocalcemia after surgery (18). 

They showed that if the level of preoperative vitamin D was less than 15 ng/ml the risk of hypocalcemia after surgery increased 15-folds, so prophylactic use of calcium and vitamin D in case of vitamin D less than 15ng/ml was recommended to reduce the rate of symptomatic hypocalcemia after total thyroidectomy (18). In other similar study by Pradeep et al. , they reported the levels of vitamin D (25OH) as one of the risk factors for postoperative hypocalcemia in 145 patients who underwent total thyroidectomy due to benign or malignant thyroid diseases (13). Contrary to these studies, there is some evidence that indicated vitamin D does not have any effect on the incidence of post-thyroidectomy hypocalcemia. Chia et al. did not find any association between serum vitamin D and post-thyroidectomy hypocalcemia (19) and in Lin’s study, although 51% of patients had vitamin D levels <30ng/ml and 20% vitamin D <20ng/ml but the low levels of vitamin D were not associated with the increased rate of postoperative hypocalcemia (20). Our research findings confirm the results of such recent studies. On the other hand, this low rate of postoperative hypocalcemia (15.2%) at the present of high rate of vitamin D deficiency (65.6%) in our study is notable, and may imply the existence of other compensatory mechanisms in the body. 

This hypothesis needs further research to determine the affecting parameters in the future. This study has a limitation regarding seasonal variations of serum vitamin D over the study period. However, in a research from geographic region of this study the authors showed no effect of seasonal variations on serum vitamin D (21). In conclusion, the results of this study indicate that vitamin D deficiency does not have a significant effect on hypocalcemia after thyroidectomy.

**Authors’ contributions**

Godazandeh, Gh: has seen and operated or on of the patients and did the postoperative follow up examinations in the hospital. Study design and Drafting the article. Kashi, Z: has done study design and drafting the article and the pre and postoperative follow up, Data collection. Godazandeh, F: has contributed to the data collection and logistics of the study and drafting the article. Tayebi, P: Data analysis, Drafting the article and final approval of the version to be submitted.

## Notes

**Funding: **This study was supported through a grant (No.90-14) from Mazandaran University of Medical Sciences, Sari, Iran

## Notes

**Conflict Interest: **The authors declare that they have no competing interests.
